# Epidemiology and Prognosis of Paraneoplastic Syndromes in Hepatocellular Carcinoma

**DOI:** 10.1155/2013/684026

**Published:** 2013-12-11

**Authors:** Pik Eu Chang, Wai Choung Ong, Hock Foong Lui, Chee Kiat Tan

**Affiliations:** ^1^Department of Gastroenterology and Hepatology, Singapore General Hospital, Singapore 169608; ^2^Gleneagles Hospital, Singapore 258500

## Abstract

*Background.* Paraneoplastic syndromes (PNS) such as hypercalcaemia, hypercholesterolaemia, and erythrocytosis have been described in hepatocellular carcinoma (HCC). *Aims.* (1) To examine the prevalence, clinical characteristics, and survival of PNS in HCC patients and (2) to evaluate the extent to which each individual PNS impacts on patient survival. *Methods.* We prospectively evaluated the prevalence, clinical characteristics, and survival of PNS among 457 consecutive HCC patients seen in our department over a 10-year period and compared them with HCC patients without PNS. *Results.* PNS were present in 127 patients (27.8%). The prevalence of paraneoplastic hypercholesterolemia, hypercalcemia, and erythrocytosis 24.5%, 5.3%, and 3.9%, respectively. Patients with PNS had significantly higher alpha-fetoprotein levels, more advanced TNM stage, and shorter survival. Among the individual PNS, hypercalcemia and hypercholesterolemia were associated with more advanced disease and reduced survival but not erythrocytosis. On multivariate analysis, the presence of PNS was not found to be an independent prognostic factor for reduced HCC survival. *Conclusion.* PNS are not uncommon in HCC and are associated with poor prognosis and reduced survival due to their association with increased tumor burden. However, they do not independently predict poor survival. Individual PNS impact differently on HCC outcome; paraneoplastic hypercalcemia in particular is associated with poor outcome.

## 1. Background

Hepatocellular carcinoma (HCC) is the fifth most common malignancy worldwide and is associated with significant mortality [1]. Symptomatic HCC patients often present with constitutional symptoms such as malaise, anorexia and weight loss in association with abdominal discomfort or an abdominal mass. Occasionally, patients may present with atypical features due to paraneoplastic manifestations of HCC. The paraneoplastic syndromes (PNS) which have been associated with HCC include hypercholesterolemia, hypercalcemia, erythrocytosis, hypoglycemia, demyelinating disease, pemphigus, polyarthritis, encephalomyelitis, and thrombocytosis [2–10]. The most common PNS associated with HCC are hypercholesterolemia, hypercalcemia, hypoglycemia, and erythrocytosis [11]. 

There is very little literature on PNS in HCC. Previous studies have suggested that HCC patients with PNS have more advanced disease, higher alpha-fetoprotein (AFP) levels, and poorer survival compared to those without [11–14]. In a study of PNS in patients with HCC in Taiwan [12], the presence of PNS was found to be an independent negative prognostic factor for survival. However, it remains unclear if a particular PNS is associated with significantly poorer outcome compared to others or whether all PNS affect outcome to a similar degree.

This study was designed to examine the prevalence and clinical characteristics of three of the most common PNS (hypercholesterolemia, hypercalcemia, and erythrocytosis) in Asian patients with HCC and to evaluate the extent to which each specific PNS impacts on patient survival.

## 2. Methodology

Clinical and laboratory data were prospectively collected from 457 patients diagnosed with HCC in the Department of Gastroenterology and Hepatology of the Singapore General Hospital between October 1988 and December 1997. The diagnosis of HCC was made based on the following criteria: (1) histology, (2) elevated serum AFP > 400 *μ*g/L and radiological features compatible with HCC (on abdominal ultrasonography, triphasic computed tomography, magnetic resonance imaging, or hepatic angiography), (3) radiological features compatible with HCC by two or more imaging modalities if AFP < 400 *μ*g/L, or (4) positive lipiodol angiography.

Clinical data collected at HCC diagnosis included gender, age at diagnosis, race, etiology of liver disease, alcohol consumption, viral serology, hematological and biochemical indices (hemoglobin, platelet counts, liver function tests, electrolytes, prothrombin time, and AFP), Child-Pugh score, and indices of severity of HCC (number of lobes involved, single versus multiple lesions, portal vein involvement, presence of metastases, and TNM stage at diagnosis). In addition, data on serum calcium levels, serum total cholesterol, triglyceride, low-density lipoprotein (LDL), and high-density lipoprotein (HDL) were collected at the time of diagnosis of HCC.

Paraneoplastic hypercalcemia was defined as the presence of hypercalcemia (corrected serum calcium greater than 2.75 mmol/L) in the absence of other known causes of hypercalcemia such as bone metastases, parathyroid disorders, and concomitant use of lithium, vitamin A, or excessive antacids. Serum parathyroid hormone (PTH) levels and bone scans to exclude metastases were performed in all patients with hypercalcemia. Paraneoplastic hypercholesterolemia was defined as fasting serum total cholesterol greater than 6.5 mmol/L, after excluding patients with known preexisting hyperlipidemia and significant cholestasis. Paraneoplastic erythrocytosis was defined as a hemoglobin level greater than or equal to 16.7 g/dL in the absence of other causes of secondary erythrocytosis such as chronic hypoxia, polycythemia rubra vera, and myeloproliferative disease. These definitions were based on the upper limit of normal in our local laboratory and are comparable to the definitions used in other publications on PNS [11–14]. This study does not include paraneoplastic hypoglycemia as we did not prospectively collect data on blood glucose levels.

Patients were followed up from time of diagnosis until May 19, 2001, when a survival census was performed with the Singapore Registry of Births and Deaths which maintains updated records of death of all Singapore citizens. Patients who died of non-HCC related causes (*n* = 12) and those who were lost to follow-up (*n* = 49) were excluded from survival analysis. The latter group comprised of foreign patients who initially sought evaluation in Singapore and subsequently returned to their home country for further treatment.

The clinical characteristics, severity of disease, and survival of HCC patients with and without PNS were analyzed. Intergroup differences were compared using Chi-square analysis (or Fisher exact test where applicable) for discrete variables and Student's *t*-test for continuous variables. Data in tables are presented as mean ± standard deviation. Survival from time of diagnosis of HCC was analyzed using the Kaplan-Meier method and compared by log-rank analysis. Univariate and multivariate Cox regression analyses were performed to identify independent prognostic factors for survival. Statistical analyses were performed using SPSS version 14 (Chicago, USA). A *P* value of less than 0.05 was considered significant. The study was approved by the institutional review board (IRB) of the hospital.

## 3. Results

Of a total of 457 patients, 127 (27.8%) were found to have at least 1 PNS (either hypercholesterolemia, hypercalcemia, or erythrocytosis). Thirteen patients (2.8%) had more than one PNS. The prevalence of paraneoplastic hypercholesterolemia, hypercalcemia, and erythrocytosis was 24.5% (112/457), 5.3%, (24/457), and 3.9% (18/457), respectively. Of 28 patients with hypercalcemia, four were diagnosed with bone metastases and were excluded from analyses of paraneoplastic hypercalcemia. Mean (±SD) corrected calcium level in the group with paraneoplastic hypercalcemia was 3.08 ± 0.22 mmol/L compared to 2.38 ± 0.16 mmol/L in those without (*P* < 0.001). Similarly, mean cholesterol level in patients with paraneoplastic hypercholesterolemia compared to those without paraneoplastic hypercholesterolemia was 8.96 ± 2.53 versus 4.43 ± 1.15 mmol/L (*P* < 0.001), and mean hemoglobin level in patients with paraneoplastic erythrocytosis compared to those without was 17.2 ± 0.6 versus 12.2 ± 2.2 g/dL (*P* < 0.001).

There was no significant difference in the age, gender, etiology of liver disease, baseline liver function, and Child-Pugh score between HCC patients with and without PNS (Table 1). To investigate whether the presence of PNS was associated with poorer prognosis, we examined its association with established indicators of poor prognosis in HCC, vis-à-vis age, serum AFP levels, bilobar involvement, multicentric disease, portal vein involvement, distant metastases, tumor stage according to the tumor, node, metastasis (TNM) classification, and Child-Pugh score at diagnosis. Amongst patients with HCC, the presence of any PNS is associated with higher AFP levels and more advanced TNM stage, indicating a poorer prognosis (Table 2).

We then examined the association of each individual PNS with the same indicators of poor prognosis (Table 3). HCC patients with paraneoplastic hypercalcemia had higher AFP levels and more advanced TNM stage. In addition, they were significantly younger and had significantly higher incidence of bilobar and multicentric disease. Paraneoplastic hypercholesterolemia was similarly strongly associated with increased AFP level but only weakly associated with advanced TNM stage. In contrast, the presence of paraneoplastic erythrocytosis was not associated with any adverse prognostic markers of HCC.

**Figure 1:**
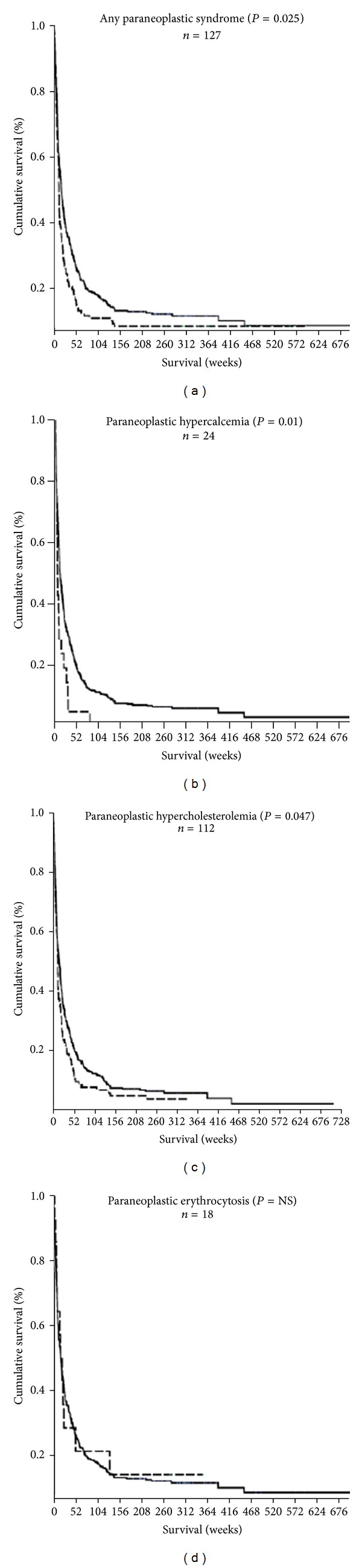
Kaplan-Meier analysis of survival in HCC patients with various paraneoplastic syndromes (dotted lines) versus HCC patients without paraneoplastic syndromes (solid lines).

Next, we studied the effect of the presence of PNS on survival in HCC. The presence of any PNS was associated with significantly reduced survival compared to HCC patients without PNS (median survival 12.4 weeks versus 18.3 weeks, resp., *P* = 0.025 by log-rank comparison). In the analysis of the association between the individual PNS and survival, we found that the survival of patients with paraneoplastic hypercalcemia and paraneoplastic hypercholesterolemia was significantly reduced compared to those without PNS (Figure 1). Median survival of patients with paraneoplastic hypercalcemia was 8.0 weeks versus 18.3 weeks in those without PNS (*P* = 0.01), and median survival of patients with paraneoplastic hypercholesterolemia was 12.9 weeks (*P* = 0.047). In contrast, survival of patients with paraneoplastic erythrocytosis was not significantly reduced.

To identify if PNS was an independent factor affecting survival in HCC, we performed a univariate analysis with the established poor prognostic factors and the presence of PNS. Nine factors (extremely elevated serum AFP levels ≥ 12,000 *μ*g/L, extent of lobar involvement, multicentric disease, portal vein involvement, presence of distant metastases, TNM stage, Child-Pugh score, presence of paraneoplastic hypercalcemia, and presence of paraneoplastic hypercholesterolemia) were identified on univariate analysis to adversely influence survival. When subjected to multivariate Cox regression analysis, AFP ≥ 12,000 *μ*g/L, portal vein involvement, bilobar disease, multicentric disease, TNM stage, and Child-Pugh grade were identified as independent negative prognostic factors for survival in HCC. However, the presence of PNS in general and specifically the presence of paraneoplastic hypercalcemia and hypercholesterolemia respectively, were not found to be independent prognostic factors for reduced survival in HCC (Table 4).

## 4. Discussion

This is the first reported study on epidemiology of PNS in HCC in South-East Asia. The prevalence of PNS in HCC in our population is 27.8%. The most common PNS is hypercholesterolemia (24.5%), followed by hypercalcemia (5.3%) and erythrocytosis (3.9%). A Medline search from 1965 to 2012 revealed few clinical studies on the prevalence and survival characteristics of PNS in HCC, mainly from East Asia [11–13] and Nigeria [14, 15]. A majority of the literature on PNS in HCC is based on isolated case reports. As such, our study with a large population of 457 patients from South-East Asia is an important contribution to the understanding of the clinical significance of PNS in HCC.

The results of our study confirm that the presence of PNS in HCC patients is associated with more advanced disease, as evidenced by significantly higher AFP levels and more advanced TNM stage at diagnosis. This reflects a greater tumor burden in patients with PNS, which in turn translates into poorer survival. These findings are congruent with earlier reports on PNS in HCC [12, 13]. Markedly elevated AFP levels reflect increased biological activity of the neoplastic hepatocytes, which are capable of producing a variety of other proteins such as erythropoietin and parathyroid hormone-related protein (PTH-RP) resulting in paraneoplastic erythrocytosis and hypercalcemia, respectively. Treatment of HCC, either via surgical resection or TACE, has been shown to abrogate PNS [16]. Paraneoplastic activity is related to overall tumor burden and biological activity of the neoplastic hepatocytes, which are significantly reduced with effective treatment of the disease and may reappear in tandem with recurrence of HCC [17].

The etiology of liver disease was not different between patients with PNS and those without. In particular, we did not find a significant difference in the prevalence of PNS between patients with HBV- and HCV-related HCC, in contrast to the findings of Luo et al. [11]. The underlying liver function and Child-Pugh scores were similar between patients with and without PNS. This suggests that the poor prognosis associated with the presence of PNS is related more to the aggressive nature of the HCC rather than to the diminished liver function.

The presence of PNS in general has been shown to be associated with poor outcome [12, 13]. However, our study shows that different individual PNS contribute to different extents towards this poor outcome. Based on our analysis, paraneoplastic erythrocytosis is not associated with a worse prognosis and does not affect survival outcome adversely. This is consistent with the findings reported in earlier studies [12, 13]. While paraneoplastic hypercholesterolemia in our patients was strongly associated with elevated AFP levels (*P* < 0.001), its association with TNM stage and its effect on survival were only just within statistical significance (*P* = 0.049 and *P* = 0.047, resp., for TNM stage and survival). In contrast, paraneoplastic hypercalcemia was strongly associated with well-recognized negative prognostic parameters of elevated AFP levels, bilobar disease, multicentric disease and TNM stage. In addition, it occurred predominantly in young patients aged 40 years and below. Importantly, it was associated with a significant reduction in survival compared to patients without PNS. Thus, paraneoplastic hypercalcemia is the most significant contributor to the poor outcome attributed to PNS in general. Furthermore, paraneoplastic hypercalcemia associated with HCC can present as a life-threatening medical emergency [18–20].

The presence of PNS in general was reported to be an independent negative prognostic factor for HCC survival based on results of a multivariate analysis [12]. In contrast, our findings demonstrated that although the presence of PNS in general was associated with poorer survival on univariate analysis, it was not found to be a significant independent negative prognostic factor when subjected to multivariate analysis. Likewise, the individual presence of paraneoplastic hypercalcemia and hypercholesterolemia, respectively, was not identified as independent negative prognostic factor for HCC survival. It is, thus, unlikely that the PNS themselves have a direct impact on the survival of the patient. The presence of PNS in HCC is associated with an increased tumor burden and more advanced disease. Such patients are often ineligible for surgical treatment (as in the case in our population), which translates to a poorer survival outcome. This may explain why PNS are associated with poorer outcome but do not independently impact on the patient's survival.

The mechanisms responsible for the various PNS remain unknown. Paraneoplastic hypercholesterolemia is believed to be due to dysregulation of LDL receptors leading to autonomous cholesterol production by neoplastic hepatocytes [21–24]. Paraneoplastic erythrocytosis is believed to occur as a result of increased tumor erythropoietin produced by the HCC or as a compensatory response to local hypoxia produced by tumor necrosis [25–27]. The etiology of paraneoplastic hypercalcemia in HCC has been attributed to the production of a variety of hormonal substances by the neoplastic hepatocytes including parathyroid hormone related peptides (PTH-RP) [28], nephrogenous cyclic AMP [29], prostaglandins [30], and tumor-related calmodulin [31].

One of the difficulties in making an accurate diagnosis of patients with PNS is the lack of standardized definitions for the various PNS. Although the data used for this research was collected more than a decade ago, these definitions remain relevant in clinical practice today. The diagnostic and staging criteria for HCC have not changed significantly in the interim period to have any meaningful impact on the parameters used in this study. However, we were not able to determine the impact of liver transplantation on survival, as it was not available in our institution during that time.

In summary, we have demonstrated that PNS are not uncommon in HCC and that while paraneoplastic syndromes in general are associated with poor prognosis and reduced survival, the individual paraneoplastic syndromes impact differently on HCC outcome. Specifically, paraneoplastic hypercalcemia is strongly associated with more advanced HCC and poor outcome, whereas paraneoplastic erythrocytosis is not associated with a poorer prognosis. However, PNS do not independently predict poor survival outcome. This study also reminds us that patients with HCC may present for the first time with atypical symptoms attributable to PNS. Therefore, in addition to assessment for the usual prognostic factors, newly diagnosed patients with HCC should also be evaluated for the presence of PNS, in particular paraneoplastic hypercalcemia.

## Figures and Tables

**Table 1:** Baseline characteristics of HCC patients with and without paraneoplastic syndromes (PNS).

Variable	HCC patients with PNS (*n* = 127)	HCC patients without PNS (*n* = 330)	*P*
Age	58.3 ± 13.5	60.8 ± 13.3	NS
Male sex	110 (86.6%)	280 (84.8%)	NS
Chinese race	112 (88.2%)	298 (90.3%)	NS
HBsAg positive	82 (64.6%)	197 (59.7%)	NS
Anti-HCV positive	6 (4.7%)	32 (9.7%)	NS
Significant alcohol intake	38 (29.9%)	97 (29.4%)	NS
Child-Pugh A/B/C	57/53/17 (44.9%/41.7%/13.4%)	146/135/49 (44.2%/40.9%/14.8%)	NS
Albumin, G/L	33.8 ± 6.3	32.7 ± 6.0	NS
Bilirubin, *μ*mol/L	43.4 ± 48.1	40.4 ± 45.6	NS
Prothrombin time, seconds	13.8 ± 2.0	14.2 ± 3.0	NS
Alanine transferase, U/L	62.9 ± 45.8	69.0 ± 100.5	NS
Aspartate transferase, U/L	137.6 ± 118.6	125.9 ± 251.2	NS

Data presented as *n* (%) or mean ± SD.

**Table 2:** Prognostic variables in HCC patients with and without paraneoplastic syndromes (PNS).

Prognostic variable	With PNS (*n* = 127)	Without PNS (*n* = 330)	*P*
Young age (≤40 years)	13 (10.2%)	28 (8.5%)	NS
AFP ≥ 12,000 *μ*g/L	59 (46.5%)	88 (26.7%)	<0.001
Bilobar disease	73 (57.5%)	158 (47.9%)	NS
Multiple lesions	92 (72.4%)	216 (65.5%)	NS
Portal vein invasion	64 (50.4%)	141 (50.9%)	NS
Distant metastases	28 (22.0%)	59 (17.9%)	NS
TNM stage (I&II/III&IV)	25/102 (19.7%/80.3%)	110/220 (33.3%/66.7%)	0.017
Child-Pugh A/B/C	57/53/17 (44.9%/41.7%/13.4%)	146/135/49 (44.2%/40.9%/14.8%)	NS

PNS: paraneoplastic syndromes; NS: not statistically significant (*P* > 0.05).

**Table 3:** Comparison of prognostic variables in HCC patients with paraneoplastic hypercalcemia, hypercholesterolemia, and erythrocytosis against those without paraneoplastic syndromes.

Prognostic variable	Without PNS	Paraneoplastic hypercholesterolemia		Paraneoplastic hypercalcemia		Paraneoplastic erythrocytosis	
	(*n* = 330)	(*n* = 112)	P*	(*n* = 24)	P*	(*n* = 18)	P*
Young age (≤40 y)	28 (8.5%)	9 (8.0%)	NS	6 (25.0%)	0.020	2 (11.1%)	NS
AFP ≥ 12,000 *μ*g/L	88 (26.7%)	54 (48.2%)	<0.001	14 (58.3%)	0.003	9 (50%)	NS
Bilobar disease	158 (47.9%)	65 (58.0%)	NS	18 (75.0%)	0.013	8 (44.4%)	NS
Multiple lesions	216 (65.5%)	82 (73.2%)	NS	22 (91.7%)	0.022	10 (55.6%)	NS
Portal vein invasion	141 (50.9%)	67 (59.8%)	NS	15 (62.5%)	NS	13 (72.2%)	NS
Distant metastases	59 (17.9%)	23 (20.5%)	NS	8 (33.3%)	NS	4 (22.2%)	NS
TNM stage (I&II/III&IV)	110/220 (33.3%/66.7%)	22/90 (19.6%/80.4%)	0.049	2/22 (8.3%/91.7%)	0.024	4/14 (22.2%/77.8%)	NS
Child-Pugh A/B/C	146/135/49 (44%/41%/15%)	48/46/18 (43%/41%/16%)	NS	9/12/3 (38%/50%/12%)	NS	11/7/0 (61%/39%/0%)	NS

PNS: paraneoplastic syndromes; NS: not statistically significant (*P* > 0.05).

P*: comparison against patients without PNS.

**Table 4:** Univariate and multivariate Cox regression analysis of prognostic factors of survival in HCC patients.

Prognostic factor	Univariate analysis			Multivariate analysis		
	*P*	Hazard ratio	95% CI*	*P*	Hazard ratio	95% CI*
AFP ≥ 12,000 *μ*g/L	<0.001	1.715	1.391–2.114	0.038	1.296	1.015–1.655
Bilobar involvement	<0.001	1.651	1.356–2.010	0.019	1.414	1.059–1.886
Multicentric tumor	0.009	1.328	1.073–1.643	0.034	1.456	1.029–2.061
Portal vein involvement	<0.001	2.129	1.711–2.649	0.002	1.507	1.159–1.960
Distant metastases	<0.001	1.628	1.278–2.074	NS	1.222	0.921–1.620
TNM stage (I&II/III&IV)	<0.001	1.964	1.508–2.558	0.047	1.546	1.006–2.377
Child Pugh Grade						
A	<0.001	1	—	<0.001	1	—
B	<0.001	1.932	1.561–2.390	<0.001	1.766	1.393–2.239
C	<0.001	2.509	1.867–3.372	0.004	1.665	1.177–2.354
Any paraneoplastic syndrome	0.025	0.782	0.630–0.970	NS	0.968	0.757–1.239
Paraneoplastic hypercalcemia	0.012	1.762	1.134–2.738	NS	1.013	0.621–1.653
Paraneoplastic hypercholesterolemia	0.013	1.335	1.062–1.677	NS	1.043	0.804–1.354
Paraneoplastic erythrocytosis	NS	—	—	—	—	—

*95% CI: 95% confidence interval, NS: not statistically significant (*P* > 0.05).
